# Notch1 signaling impairs regulatory T cells during multisystem inflammatory syndrome in children

**DOI:** 10.1172/JCI166016

**Published:** 2023-01-03

**Authors:** Magali Noval Rivas, Moshe Arditi

**Affiliations:** 1 Department of Pediatrics, Division of Infectious Diseases and Immunology, Guerin Children’s,; 2 Infectious and Immunologic Diseases Research Center (IIDRC), Department of Biomedical Sciences, and; 3 Smidt Heart Institute, Cedars-Sinai Medical Center, Los Angeles, California, USA.

## Abstract

Multisystem inflammatory syndrome in children (MIS-C) is a rare pediatric inflammatory disorder characterized by immune cell hyperactivation, cytokine storm, and the production of autoantibodies. The mechanisms underlying such immune dysregulation still need to be unraveled. In this issue of the *JCI*, Benamar et al. demonstrated the critical role of the Notch receptor 1/CD22 (Notch1/CD22) axis in Tregs, which, when activated, impairs Treg functions and promotes inflammation. They showed that the Notch1/CD22 axis contributed to dysregulated immune responses in MIS-C. These findings may have implications for MIS-C and many other inflammatory diseases.

## Multisystem inflammatory syndrome in children

In adults, COVID-19 can result in severe and potentially lethal respiratory complications associated with dysregulated and robust inflammatory responses. Children with COVID-19 are usually asymptomatic or have less severe disease than adults, and respiratory complications are unusual (1). Such differences in disease severity may arise from the distinct makeup of adult and pediatric immune responses. Innate immune responses in children appear more robust and may promote better viral clearance, whereas the adaptive immune response is more naive and less cytotoxic, which could contribute to the milder disease observed in these patients (2).

However, in late April 2020, an infectious febrile pediatric inflammatory disorder called multisystem inflammatory syndrome in children (MIS-C) was first reported (3, 4). MIS-C develops in previously healthy children two to five weeks after mild or asymptomatic SARS-CoV-2 infection (1). Affected children present with fever and high levels of biomarkers indicative of hyperinflammation and potential cardiovascular complications (3, 4). MIS-C may lead to cardiogenic shock and multiorgan failure, requiring intensive care unit admission.

## Dysregulated immune responses in patients with MIS-C

Immune profiling of patients with MIS-C has revealed immune dysregulation, characterized by increased activation of innate and adaptive immune cells and intense release of cytokines and chemokines (5-9). Indeed, patients with MIS-C have elevated levels of IFN-γ, IL-6, IL-17A, TNF-α, and IL-1β (6, 8, 9). Neutrophils, NK cells, and monocytes are activated, and they upregulate cytotoxic genes and alarmins during MIS-C (5, 10, 11). Patients with MIS-C present with lymphopenia and higher frequencies of activated and proliferating T cells (6, 7, 10, 11). Several studies have reported the expansion of Vβ11-2^+^ T cells in these patients, which correlates with inflammatory markers and MIS-C severity (8–10, 12). Computational analysis predicts that these expanding Vβ11-2^+^ T cells will strongly interact with a superantigen-like motif previously identified in the SARS-CoV-2 spike 1 glycoprotein (12–14). Increased plasmablast frequencies and detection of autoantibodies targeting ubiquitously expressed antigens and self-antigens in patients with MIS-C further implicate a dysregulated autoinflammatory component in MIS-C pathogenesis (5–7, 10, 15).

It remains unclear today why only a fraction of children exposed to SARS-CoV-2 develop this rare hyperinflammatory syndrome and which factors influence MIS-C susceptibility. Interestingly, a combination of three HLA class I alleles (A02, B35, and C04) has been reported in patients with severe MIS-C and Vβ11-2^+^ T cells expansion, but not in patients with mild MIS-C without Vβ11-2^+^ T cell expansion (12). Furthermore, rare genetic variants involved in the regulation of inflammatory responses are also present in some patients with MIS-C (16). Altogether, these findings indicate the presence of dysregulated immune responses during MIS-C, but we still lack a complete understanding of the cellular and molecular mechanisms leading to such immune dysregulation.

## Tregs and Notch receptors in inflammation

Tregs are crucial for immune homeostasis. Through their suppressive capacities, Tregs promote immune tolerance and limit excessive effector immune responses that may become pathogenic in the host. Dysfunctional or dysregulated Treg responses result in the development of autoimmune and inflammatory diseases. Notch receptors are involved in several biological processes, including the regulation of immune responses. In particular, Notch signaling on T cells induces their activation, proliferation, differentiation, and cytokine production. Circulating Tregs from adult patients with COVID-19 exhibit upregulated Notch receptor 4 (Notch4) expression, which increases with disease severity (17). In murine viral infection models, Notch4 expression by Tregs impairs their function and tissue repair capacities (17).

Dysfunctional Tregs could contribute to the hyperinflammation observed in patients with MIS-C. In a longitudinal study of a cohort of patients with MIS-C, while the proportion of activated Tregs (HLA-DR^+^) remained unchanged over time, Treg counts appeared to increase at the resolution and convalescence phase of the disease (11). Sacco et al. reported that in the early phase of MIS-C, the levels of CCL22, a chemokine known to promote Treg migration and function, are decreased (9). Low levels of CCL22 may contribute to reduced Treg responses and uncontrolled inflammatory responses during MIS-C (9). While Notch4 signaling on Tregs contributes to severe COVID-19 in adult patients, whether activation of Notch signaling pathways on Tregs promotes dysregulated immune responses during MIS-C is unclear. Insights into the Treg compartment and the functionality of these cells during MIS-C are currently lacking.

## Upregulation of the Notch1/CD22 axis on Tregs

**Figure 1:**
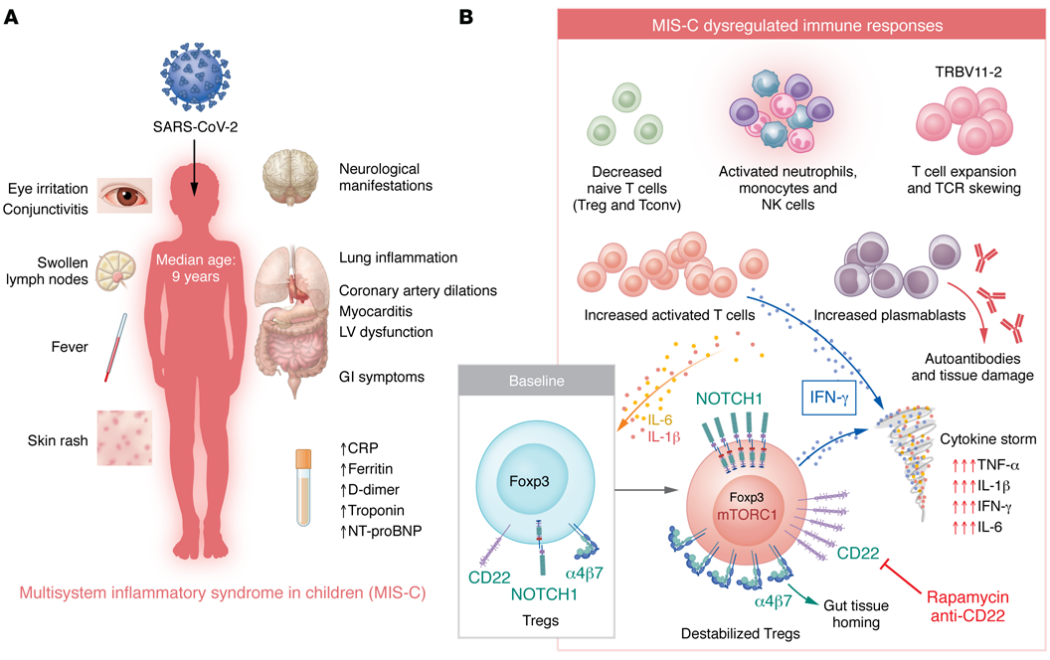
Immune responses are dysregulated during MIS-C. (**A**) MIS-C develops in children two to five weeks after SARS-CoV-2 infection or exposure. Patients present with fever and elevated markers of inflammation associated with gastrointestinal (GI), cardiovascular, and neurological manifestations. (**B**) MIS-C is characterized by increased activation of neutrophils, monocytes, and NK cells. Decreased proportions of naive T cells (CD4^+^ Tconv and Tregs) and increased proportions of activated cells are also reported during MIS-C. Furthermore, TCR repertoire skewing and an expansion of TRBV11-2 T cell clonotypes, increased frequencies of plasmablasts, and the presence of autoantibodies have also been reported during MIS-C. In this issue of the *JCI*, Benamar et al. show that MIS-C was also associated with dysregulated Treg responses. In the presence of IL-1β and IL-6, Tregs from patients with MIS-C upregulated the expression of Notch1, CD22, and the gut-homing integrin α4β7. Activation of the Notch1/CD22 pathway in Tregs resulted in their instability, decreased Foxp3 expression, and subversion toward IFN-γ–producing effector cells, further propagating the MIS-C hyperinflammatory response. Blocking CD22 or using rapamycin, an mTORC1 inhibitor, inhibited the Notch1/CD22-triggered dysregulation of Tregs (18). CRP, C-reactive protein; LV, left ventricular; NT-proBNP, N-terminal pro-brain natriuretic peptide.

In this issue of the *JCI*, Benamar et al. unraveled a critical role of the Notch1/CD22 signaling axis on Tregs in MIS-C pathogenesis (18) (Figure 1). By elegantly combining an analysis of samples collected from patients before and after treatment with an analysis of murine models in which Notch1 activation on Tregs was manipulated in vivo, the authors go beyond previously published studies profiling MIS-C inflammatory responses and provide mechanistic insights into immune homeostasis breakdown and hyperinflammation development in MIS-C (18).

First, Benamar et al. analyzed the CD4^+^ T cell compartment of patients with MIS-C before and after treatment and confirmed the increased activation of conventional CD4^+^ T (Tconv) cells before treatment. Prior to treatment, Tregs were largely destabilized and showed activation of the mTORC1 pathway and upregulated expression of Notch1 (18). In vitro, IL-1β or IL-6, which are produced at high levels during MIS-C, enhanced Notch1 expression on activated Tregs (18). Additionally, Tregs from patients with acute MIS-C showed a selective increase in the production of IFN-γ. These observations suggest that Notch1 signaling on Tregs during MIS-C may destabilize and functionally subvert them from their suppressive functions.

Second, the authors identified dominant-negative loss-of-function rare mutations in *NUMB* and *NUMBL* genes in some patients with MIS-C. These two genes negatively regulate Notch receptor signaling, and Tregs from patients with MIS-C harboring the *NUMB* or *NUMBL* mutations had upregulated Notch1 expression (18). Tregs from patients with MIS-C expressing Notch1 also exhibited higher levels of CD22, a protein known to control B cell receptor signaling and B cell homing to intestinal tissues through upregulation of the gut-homing receptor α4β7 (18). Benamar et al. observed increased expression of α4β7 in circulating Tregs from patients with MIS-C, hinting that these cells may migrate to intestinal mucosal tissues. The authors further showed that the dysfunction of Tregs from patients with MIS-C was CD22 dependent, as the suppressive capacities of these Tregs could be restored upon treatment with an anti-CD22 mAb in vitro (18).

Finally, the authors provided in vivo mechanistic insights into the Notch1/CD22 axis and dysregulated immune responses using two transgenic mouse models, both of which resulted in increased Notch1 activity specifically in Tregs. To mimic viral infection, mice were injected with polyinosinic:polycytidylic acid (poly I:C) (18). Notably, activation of the Notch1 pathway in Tregs recapitulated the phenotype observed in patients with MIS-C, specifically, increased activation of Tconv cells and Tregs, upregulation of α4β7 and CD22 by Tregs, and destabilization of Tregs, as demonstrated by their heightened production of IFN-γ (18). Furthermore, treatment with an anti-CD22 mAb decreased this poly I:C–induced multiorgan inflammatory phenotype (18). By enhancing T cell receptor (TCR) signaling in Tregs, CD22 destabilized and impaired Treg-suppressive functions in a mTORC1-dependent manner, which was reversible with either anti-CD22 mAb or rapamycin, an mTORC1 inhibitor (18).

## Conclusions and future directions

Several exciting discoveries emerge from the study by Benamar and colleagues that improve our understanding of the cellular and molecular immune mechanisms underlying MIS-C development. This study indicates that therapies targeting either CD22 or mTORC1 might be useful for patients with MIS-C who are resistant to standard-care antiinflammatory therapy. The incidence of MIS-C has steeply decreased over the past year, which may be the result of variant mutations in the SARS-CoV-2 spike protein associated with less severe disease as well as of pediatric vaccination, which is effective in preventing MIS-C (19). However, it remains unclear why only a fraction of SARS-CoV-2–exposed children developed this syndrome, how immune homeostasis breaks down during MIS-C, and why this syndrome appears weeks after initial viral exposure. Hence, it is still necessary to characterize the cellular and molecular mechanisms leading to this delayed and uncontrolled hyperinflammatory response. The study by Benamar et al. (18) supports the concept that a systemic spread of inflammation involves the mobilization of tissue-specific, Treg-dependent licensing mechanisms. In MIS-C, the authors found that the Notch1/CD22 axis destabilized Tregs, subverted their regulatory functions, and further licensed the systemic spread and gut inflammation characteristic of this disease (18). These observations complement previously published studies indicating overactivation of immune cells, autoimmune responses, and TCR repertoire skewing suggestive of activation by the superantigen-like motif identified in SARS-CoV-2. The knowledge gained from this study extends beyond MIS-C and may impact our understanding of other hyperinflammatory syndromes.

## Version 1 01/03/2023

Electronic publication

## References

[1] Chou J (2022). Immunology of SARS-CoV-2 infection in children. Nat Immunol.

[2] Pierce CA (2022). COVID-19 and children. Science.

[3] Riphagen S (2020). Hyperinflammatory shock in children during COVID-19 pandemic. Lancet.

[4] Verdoni L (2020). An outbreak of severe Kawasaki-like disease at the Italian epicentre of the SARS-CoV-2 epidemic: an observational cohort study. Lancet.

[5] Gruber CN (2020). Mapping systemic inflammation and antibody responses in multisystem inflammatory syndrome in children (MIS-C). Cell.

[6] Consiglio CR (2020). The immunology of multisystem inflammatory syndrome in children with COVID-19. Cell.

[7] Vella LA (2021). Deep immune profiling of MIS-C demonstrates marked but transient immune activation compared to adult and pediatric COVID-19. Sci Immunol.

[8] Moreews M (2021). Polyclonal expansion of TCR Vbeta 21.3^+^ CD4^+^ and CD8^+^ T cells is a hallmark of multisystem inflammatory syndrome in children. Sci Immunol.

[9] Sacco K (2022). Immunopathological signatures in multisystem inflammatory syndrome in children and pediatric COVID-19. Nat Med.

[10] Ramaswamy A (2021). Immune dysregulation and autoreactivity correlate with disease severity in SARS-CoV-2-associated multisystem inflammatory syndrome in children. Immunity.

[11] Carter MJ (2020). Peripheral immunophenotypes in children with multisystem inflammatory syndrome associated with SARS-CoV-2 infection. Nat Med.

[12] Porritt RA (2021). HLA class I-associated expansion of TRBV11-2 T cells in multisystem inflammatory syndrome in children. J Clin Invest.

[13] Cheng MH (2020). Superantigenic character of an insert unique to SARS-CoV-2 spike supported by skewed TCR repertoire in patients with hyperinflammation. Proc Natl Acad Sci U S A.

[14] Noval Rivas M (2022). Multisystem inflammatory syndrome in children and long COVID: The SARS-CoV-2 viral superantigen hypothesis. Front Immunol.

[15] Porritt RA (2021). The autoimmune signature of hyperinflammatory multisystem inflammatory syndrome in children. J Clin Invest.

[16] Chou J (2021). Mechanisms underlying genetic susceptibility to multisystem inflammatory syndrome in children (MIS-C). J Allergy Clin Immunol.

[17] Harb H (2021). Notch4 signaling limits regulatory T-cell-mediated tissue repair and promotes severe lung inflammation in viral infections. Immunity.

[18] Benamar M (2022). The Notch1/CD22 signaling axis disrupts Treg function in SARS-CoV-2–associated multisystem inflammatory syndrome in children. J Clin Invest.

[19] Zambrano LD (2022). Effectiveness of BNT162b2 (Pfizer-BioNTech) mRNA vaccination against multisystem inflammatory syndrome in children among persons aged 12–18 Years - United States, July-December 2021. MMWR Morb Mortal Wkly Rep.

